# TRPM8 as an Anti–Tumoral Target in Prostate Cancer Growth and Metastasis Dissemination

**DOI:** 10.3390/ijms23126672

**Published:** 2022-06-15

**Authors:** Guillaume P. Grolez, Giorgia Chinigò, Alexandre Barras, Mehdi Hammadi, Lucile Noyer, Kateryna Kondratska, Etmar Bulk, Thibauld Oullier, Séverine Marionneau-Lambot, Marilyne Le Mée, Stéphanie Rétif, Stéphanie Lerondel, Antonino Bongiovanni, Tullio Genova, Sébastien Roger, Rabah Boukherroub, Albrecht Schwab, Alessandra Fiorio Pla, Dimitra Gkika

**Affiliations:** 1 Laboratoire de Physiologie Cellulaire, INSERM U1003, Laboratory of Excellence, Ion Channels Science and Therapeutics, University of Lille, 59000 Villeneuve d’Ascq, France; grolez.guillaume@gmail.com (G.P.G.); giorgia.chinigo@unito.it (G.C.); lucile.noyer@inserm.fr (L.N.); katiushonok@yahoo.com (K.K.); alessandra.fiorio@unito.it (A.F.P.); 2 Department of Life Science and Systems Biology, University of Turin, 10123 Turin, Italy; tullio.genova@gmail.com; 3 CNRS, Centrale Lille, Univ. Lille, Univ. Polytechnique Hauts-de-France, UMR 8520—IEMN, 59000 Lille, France; alexandre.barras@univ-lille.fr (A.B.); mehdi.h28@gmail.com (M.H.); rabah.boukherroub@univ-lille.fr (R.B.); 4 Institute of Physiology II, University of Münster, 48149 Münster, Germany; ebulk@uni-muenster.de (E.B.); aschwab@uni-muenster.de (A.S.); 5 Cancéropôle du Grand Ouest, Plateforme In Vivo, 44000 Nantes, France; thibauld.oullier@inserm.fr (T.O.); severine.marionneau-lambot@inserm.fr (S.M.-L.); 6 CNRS UAR44, PHENOMIN-TAAM, 45071 Orléans, France; lemee@cnrs-orleans.fr (M.L.M.); stephanie.retif@cnrs-orleans.fr (S.R.); lerondel@cnrs-orleans.fr (S.L.); 7 CNRS, Inserm, CHU Lille, Institut Pasteur de Lille, US 41—UMS 2014—PLBS, University of Lille, 59000 Lille, France; antonino.bongiovanni@univ-lille.fr; 8 Nanostructured Interfaces and Surfaces Centre of Excellence (NIS), University of Turin, 10123 Turin, Italy; 9 Transplantation, Immunologie et Inflammation T2I-EA 4245, Université de Tours, 37044 Tours, France; sebastien.roger@univ-tours.fr; 10 CNRS, INSERM, CHU Lille, Centre Oscar Lambret, UMR 9020-UMR 1277-Canther-Cancer Heterogeneity, Plasticity and Resistance to Therapies, University Lille, 59000 Villeneuve d’Ascq, France; 11 Department of Molecular and Cell Biology, University of California, Berkeley, CA 94720, USA; 12 Institut Universitaire de France (IUF), 75231 Paris, France

**Keywords:** prostate cancer, tumor growth, metastasis dissemination, TRPM8, Rho signaling, ERK, FAK, cell proliferation, trans–endothelial migration, invasion

## Abstract

In the fight against prostate cancer (PCa), TRPM8 is one of the most promising clinical targets. Indeed, several studies have highlighted that TRPM8 involvement is key in PCa progression because of its impact on cell proliferation, viability, and migration. However, data from the literature are somewhat contradictory regarding the precise role of TRPM8 in prostatic carcinogenesis and are mostly based on in vitro studies. The purpose of this study was to clarify the role played by TRPM8 in PCa progression. We used a prostate orthotopic xenograft mouse model to show that TRPM8 overexpression dramatically limited tumor growth and metastasis dissemination in vivo. Mechanistically, our in vitro data revealed that TRPM8 inhibited tumor growth by affecting the cell proliferation and clonogenic properties of PCa cells. Moreover, TRPM8 impacted metastatic dissemination mainly by impairing cytoskeleton dynamics and focal adhesion formation through the inhibition of the Cdc42, Rac1, ERK, and FAK pathways. Lastly, we proved the in vivo efficiency of a new tool based on lipid nanocapsules containing WS12 in limiting the TRPM8–positive cells’ dissemination at metastatic sites. Our work strongly supports the protective role of TRPM8 on PCa progression, providing new insights into the potential application of TRPM8 as a therapeutic target in PCa treatment.

## 1. Introduction

In Western industrialized countries, prostate cancer (PCa) is the most frequently diagnosed cancer and the second most common cause of death among men [1]. In its first stages, PCa develops slowly and remains localized, while in later stages, the prostate capsule barrier can be crossed and the tumor becomes invasive, often leading to metastasis in the lymph nodes and, later, mainly in the bones, liver, and lungs [2].

Since metastases are the leading cause of cancer–related death, the deepest knowledge about the mechanisms supporting cancer metastasis is crucial in the fight against cancer. Malignant cell transformation is the result of enhanced proliferation, aberrant differentiation, and an impaired ability to die [3]. These alterations ultimately result in abnormal tissue growth, which can eventually turn into the uncontrolled expansion and invasion characteristic of cancer. Moreover, to escape from the primary site and colonize distant organs, cancer cells must acquire a more aggressive migratory and invasive phenotype. In this context, several studies have pinpointed the central role played by calcium (Ca^2+^) and ion channels in all the processes associated with the metastatic cascade [4,5,6,7]. In particular, members of the Transient Receptor Potential (TRP) ion channel superfamily were implicated in all the hallmarks of cancer, and their expression levels correlated with the emergence and/or progression of numerous epithelial cancers [8,9]. In addition, it should be noted that besides their transcriptional and translational regulation, ion channel trafficking to the cell surface, as well as plasma membrane stabilization, define channel activity. Therefore, modulation of TRP channel expression/activity on one of these levels may affect intracellular Ca^2+^ concentrations and, consequently, processes involved in carcinogenesis, such as proliferation, apoptosis, and migration [10].

In recent decades, several lines of evidence have highlighted the key involvement of the TRP channel TRPM8 in prostate cancer. First, TRPM8 expression varies during cancer progression, with strong expression in the initial PCa stages and loss of expression in the late and more aggressive stages. This differential expression makes TRPM8 a good diagnostic/prognostic marker and, indeed, it is currently used as a clinical marker in some countries [11,12]. Second, several in vitro studies have reported anti–proliferative [13,14,15], pro–apoptotic [15,16], and anti–migratory [15,17,18] effects of TRPM8 in PCa cells. However, although an anti–proliferative role was suggested for TRPM8 in vivo [14], other studies have reported a pro–proliferative role in vitro [16,19,20]. More precisely, these in vitro studies reported a decrease in the proliferation rate in several PCa cell lines when TRPM8 was inhibited by pharmacological blockers and, to a lesser extent, by siRNA against the channel. Indeed, TRPM8 blockers and siRNA reduced the proliferation in LNCaP and DU–145 cells, but not in PC3 cells or PNTA1 cells. Taking into account the broad action of pharmacological blockers and the uncorrelated effects between TRPM8 knock–down expression and cell proliferation, it is difficult to speculate on the physiological relevance of the channel in that case. These data, in addition to the antitumorigenic effect of TRPM8 observed in a subcutaneous xenograft model in mice [14], illustrate the need for further studies to better confirm and clearly delineate the anti–metastatic role played by TRPM8 in PCa progression.

To shed light on these contradictory data and further support the protective role of TRPM8 in vivo, in this study, we explored the effects of TRPM8 overexpression on PCa progression both in vitro and in vivo using a prostate orthotopic xenograft mouse model. In particular, we evaluated the impact of TRPM8 on primary tumor growth and metastatic dissemination, further investigating the molecular mechanisms underlying TRPM8′s effects on PCa progression. These findings may provide new insight in view of a potential application of TRPM8 as a therapeutic target in the treatment of PCa using channel–targeting nanotechnologies.

## 2. Results

### 2.1. TRPM8 Overexpression Inhibits Prostate Tumor Growth In Vivo

**Figure 1:**
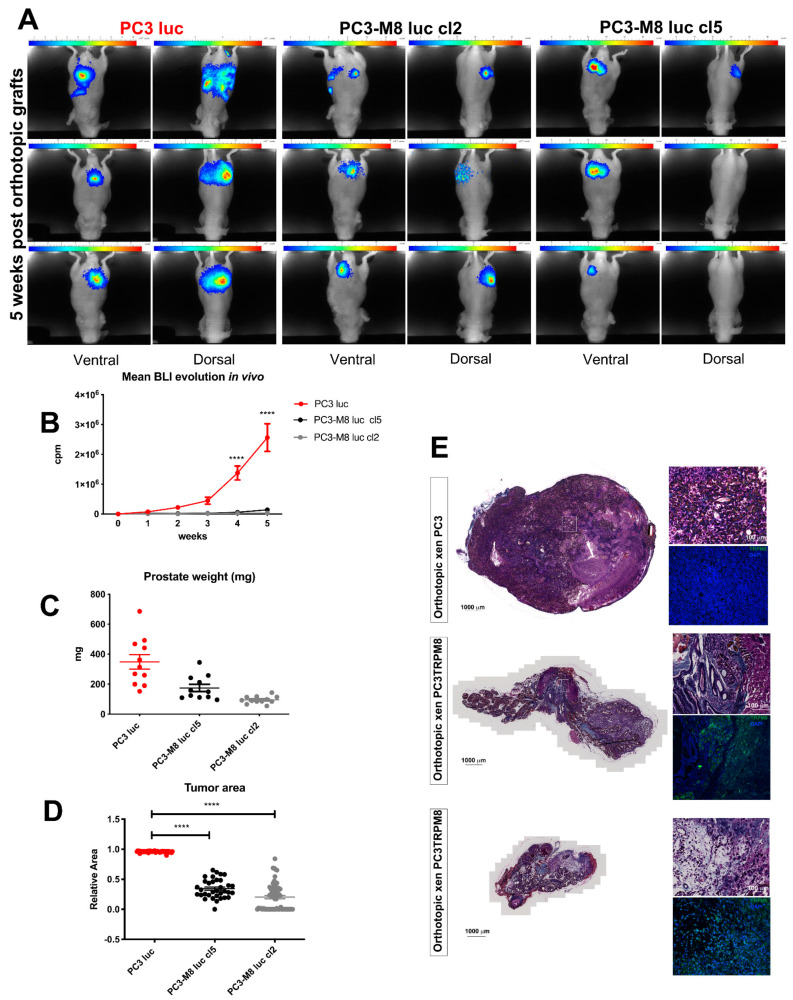
TRPM8 overexpression inhibits prostate primary tumor growth in vivo. (**A**) Images showing bioluminescence of mice grafted with PC3 luc and two clones of TRPM8–overexpressing PC3–M8 luc cells (cl2 and cl5) 5 weeks after orthotopic graft. (**B**) Quantification of bioluminescence from PC3 luc (red line); and PC3–M8 luc (black and grey lines); mice xenografts 5 weeks after grafting (n = 12 mice/condition; statistical significance **** = *p* <0.00001, two–way ANOVA with post hoc Bonferroni test. (**C**) Weight (mg) of prostate tumors of mice 5 weeks after grafting with PC3 luc or PC3–M8 luc (n = 12 mice/condition). (**D**) Quantification of the relative tumor area from PC3 luc (red dots)and PC3–M8 luc grafts in mice (n = 51 for PC3 luc; n = 34 for PC3–M8 luc cl5 (black dots), and n = 63 for PC3–M8 luc cl2 (grey dots); statistical significance: **** = *p* < 0.00001, ordinary one–way ANOVA with post hoc Tukey’s test). (**E**) Histological analysis of tumors from PC3 luc and PC3–M8 luc mouse xenografts. Immunofluorescence for TRPM8 is represented in green, and immunofluorescence for DAPI is represented in blue.

To investigate the role played by TRPM8 in prostate tumor growth, we performed an in vivo study using mice with a prostate orthotopic graft of PCa cells from bone metastases stably overexpressing (PC3–M8 luc) or not expressing (PC3 luc) the TRPM8 channel. We used two separate clones, named cl2 and cl5, for the PC3–M8 luc assays. These clones were obtained after the transfection of PC3–M8 cells with a pcdna–3 luciferase plasmid and then selected using geneticin. Bioluminescence based on the luciferase activity was measured weekly to follow PC3 cell growth, and we showed that the overexpression of TRPM8 inhibited prostate tumor growth (Figure 1A,B). More precisely, the overexpression of TRPM8 induced a significant decrease in PCa growth, which was already evident 4 weeks after the orthotopic graft and was equal to 93.34 ± 1.81% in PC3–M8 luc cl5 and 98.04 ± 0.48% in PC3–M8 luc cl2 5 weeks later (Figure 1A,B).

Following mouse sacrifice, prostate tumors were recovered, weighed, and analyzed (Figure 1C–E). In accordance with bioluminescence measurements, we found that TRPM8 overexpression decreased the tumor weight by 42.89 ± 7.73% in PC3–M8 luc cl5 grafts (175.08 ± 23.90 mg for PC3–M8 luc cl5 tumors versus 348.6 ± 48.52 mg for PC3 luc tumors) and by 66.31 ± 5.79% in PC3–M8 luc cl2 grafts (95.83 ± 6.99 mg for PC3–M8 luc cl2 tumors versus 348.6 ± 49.52 mg for PC3 luc tumors) (Figure 1C). The comparison of the tumor area between PC3 luc and PC3–M8 luc grafts also confirmed the inhibitory role of TRPM8 in PCa growth, showing a reduction in the tumor area of 79.91 ± 3.28% in PC3–M8 luc cl5 and 64.24 ± 3.41% in PC3–M8 luc cl2 (Figure 1D). Immunofluorescence and histological analyses were further performed to verify TRPM8 expression in our prostate orthotopic tumor model (Figure 1E). As shown in Figure 1E, the prostatic glandular architecture of the PC3 luc orthotopic grafts was severely disorganized compared with that of the TRPM8 grafts.

### 2.2. TRPM8 Overexpression Inhibits Cell Proliferation and Cell Clone Formation

**Figure 2:**
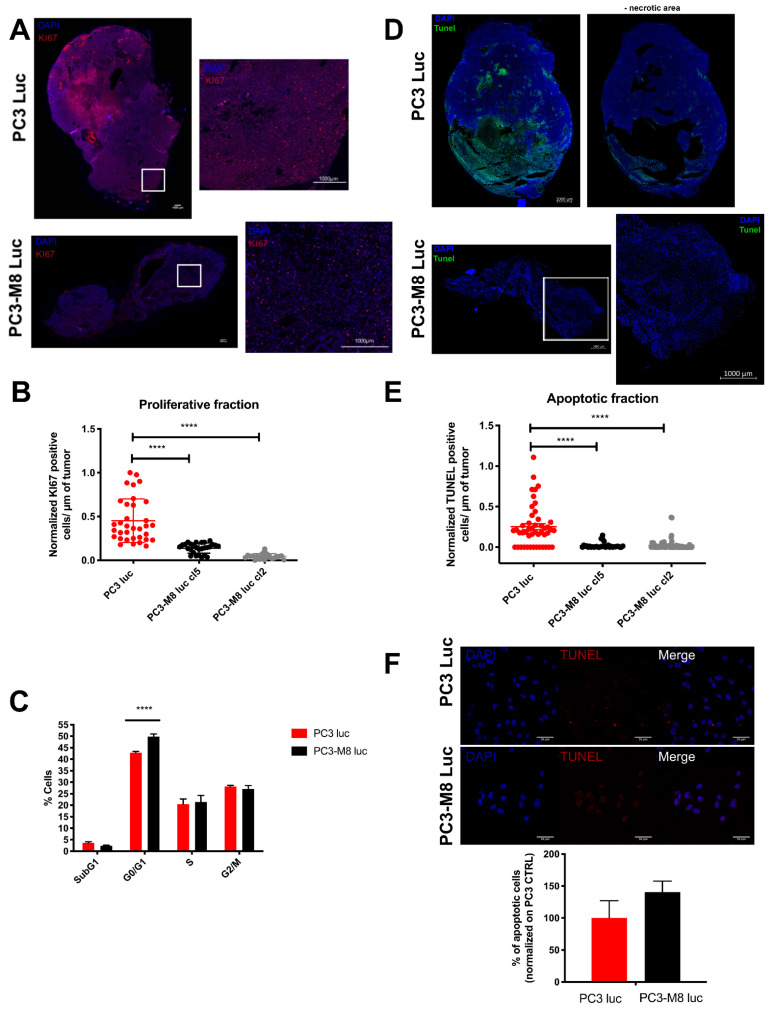
TRPM8 overexpression affects cell proliferation but not apoptosis. (**A**) Representative images of Ki67 staining in prostate tumors from PC3 luc and PC3–M8 luc orthotopic grafts. (**B**) Quantification of Ki67 staining of tumors from PC3 luc and PC3–M8 luc grafts. Data are expressed as Ki67 positive cells/µm (n = 34 for PC3 luc (red dots); n = 28 for PC3–M8 luc cl5 (black dots); and n = 21 for PC3–M8 luc cl2 (grey dots); statistical significance: **** = *p* < 0.0001, ordinary one–way ANOVA with post hoc Tukey’s test). (**C**) Flow cytometry assay with propidium iodide on PC3 luc (red dots); PC3–M8 luc cl5 (black dots) and PC3–M8 luc cl2 (grey dots) (n = 5 independent assays; statistical significance: **** = *p* <0.0001, two–way ANOVA with post hoc Sidak test). (**D**) Images representing apoptotic fraction (green signal) in tumors from PC3 luc and PC3–M8 luc grafts (necrotic areas were subtracted on tumors from PC3 luc grafts). Cells were labeled with DAPI (blue signal). (**E**) Quantification of apoptotic area in tumors from PC3 luc and PC3–M8 luc grafts. Data are expressed as TUNEL positive cells/µm (n = 30 images/condition; statistical significance: **** = *p* < 0.0001, ordinary one–way ANOVA with post hoc Tukey’s test). (**F**) Apoptosis assay of PC3 luc and PC3–M8 luc cell lines using TUNEL kit.

To better clarify the mechanism underlying the TRPM8–mediated inhibition of PCa growth observed in vivo, we performed histological and immunofluorescence analyses of prostatic tissues extracted from mouse xenografts. In particular, we quantified proliferation and apoptotic markers that could account for the large difference observed between the PC3 luc and PC3–M8 luc orthotopic grafts (Figure 2). The proliferation rate in xenografted mice prostates was assessed by KI67 staining, which showed that TRPM8 overexpression significantly decreased the proliferative fraction of PC3 cells by 55.70 ± 7.87% and 88.20 ± 15.87% in PC3–M8 luc cl 5 and 2, respectively (Figure 2A,B). Consistently, TRPM8 overexpression in PC3 cells revealed cell cycle arrest at the G_0_/G_1_ transition, with an increase of 6.97 ± 1.21% for PC3–M8 luc cells in the G_0_/G_1_ phase compared with PC3 luc cells in vitro (Figure 2C). However, this percentage of cell cycle arrest is too limited to explain the large difference found in tumor size and volume due to TRPM8 overexpression (Figure 1). Therefore, we next evaluated apoptosis in the cancerous prostates of mice using the TUNEL assay. As shown in Figure 2D, PC3 luc grafts, unlike PC3–M8 luc grafts, included apoptotic and necrotic areas. In agreement, TUNEL quantification ex vivo revealed a significant decrease of 95.44 ± 14.48% in the apoptotic area in PC3–M8 luc tumors from both clones compared with PC3 luc tumors (Figure 2D). However, the same assay performed in vitro on PC3 cells revealed that TRPM8 overexpression did not induce a significant difference in the number of apoptotic cells (Figure 2F). This evidence suggests that TRPM8 prevents prostate tumor growth by reducing the proliferation rate without inducing apoptosis.

**Figure 3:**
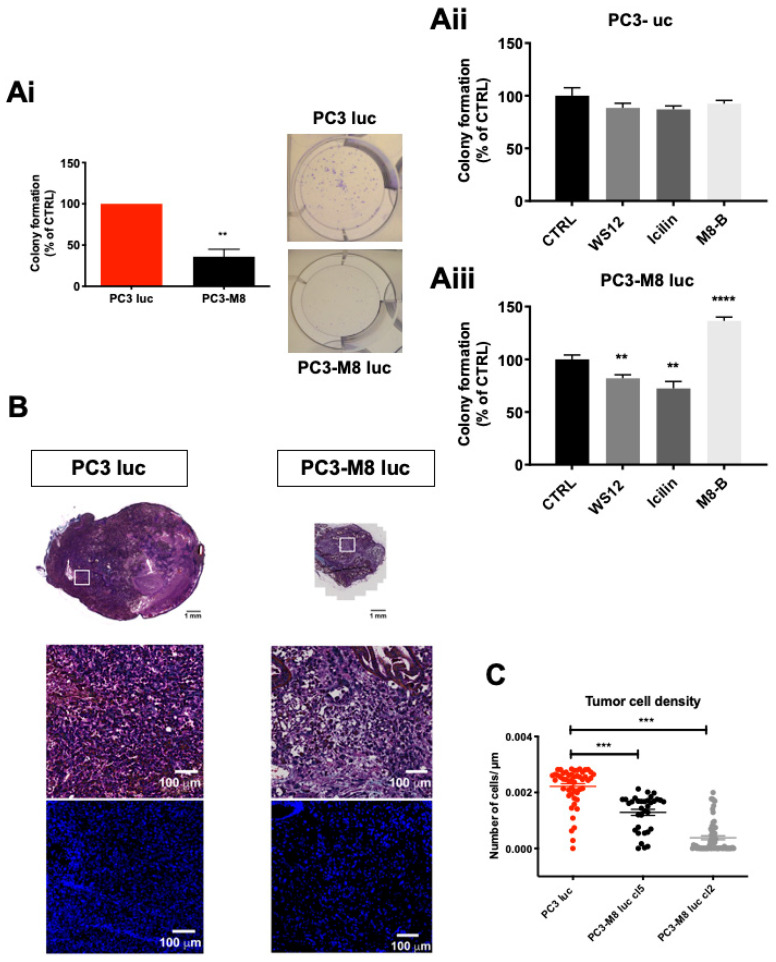
TRPM8 overexpression inhibits clone formation capability. (**A**) In vitro clonogenic assay of PC3 luc and PC3–M8 luc. Cells were seeded at 5 × 10^4^ cells/well for two weeks and treated (**Aii**,**Aiii**) or not treated (**Ai**) with WS–12 (100 nM), icilin (10 µM), or M8–B (1 µM). The number of clones was quantified and normalized in the control condition (n = 6 independent experiments; statistical significance: ** = *p* < 0.01, **** = *p* < 0.001, unpaired Student’s *t*–test). (**B**) Images representing DAPI staining in tumors from PC3 luc and PC3–M8 luc grafts. (**C**) Quantification of cell density in tumors from PC3 luc (red dots); PC3–M8 luc cl5 (black dots) and PC3–M8 luc cl2 (grey dots) grafts. Tumor cell density is expressed as number of cell/µm^2^ (n = 50 images/condition; statistical significance: *** = *p* < 0.001, ordinary one–way ANOVA with post hoc Tukey’s test).

Beyond proliferation and apoptosis, cells’ capability to form clones plays a crucial role in tumor growth. We thus performed clonogenic assays with PC3 luc and PC3–M8 luc (Figure 3A). As shown in Figure 3(Ai), TRPM8 overexpression led to a significant decrease (by 64.12 ± 8.99%) in the cellular capacity to form clones. Moreover, TRPM8 stimulation with WS12 (10 nM) or icilin (10 µM) further compromised PC3–M8 luc cells’ capability to form clones (by 17.97 ± 3.40% and 27.51 ± 6.61%, respectively, Figure 3(Aiii)), whereas no effects were detected on PC3 luc control cells after agonist treatment (Figure 3(Aii)). Accordingly, TRPM8 inhibition with the M8B (1 µM) antagonist restored the capability of PC3–M8 luc to form clones and increased it by 36.27 ± 3.52% (Figure 3(Aiii)). To further support these in vitro data, analyses of tumors from PC3 luc and PC3–M8 luc orthotopic grafts by DAPI staining were performed. As shown in Figure 3B, a higher density of PC3 luc cells was detected compared with that of PC3–M8 luc cells in tumor areas (Figure 3B). More precisely, cell density quantification performed tumor–by–tumor revealed that, in PC3 luc grafts, the cell density was 82.80 ± 14.80% higher than that in PC3–M8 luc cl5 grafts and 41.95 ± 3.27% higher than that in tumors from PC3–M8 luc cl2 grafts (2.42 × 10^−3^ cells/µm^2^ in PC3 luc tumors, 3.80 × 10^−4^ cells/µm^2^ in PC3–M8 luc cl5 tumors, and 1.28 × 10^−3^ cells/µm^2^ in PC3–M8 luc cl2 tumors) (Figure 3C). Collectively, our data on PC3 luc and PC3–M8 luc cell lines in vitro as well as in xenografted mouse prostates demonstrate that the decrease in tumor growth induced by TRPM8 overexpression (Figure 1) is partly attributable to the TRPM8–induced cell cycle arrest at the G_0_/G_1_ phase as well as its strong inhibition of PCa cells’ clone formation capabilities.

### 2.3. TRPM8 Overexpression Inhibits PCa Cell Dissemination In Vivo and Constitutes an Efficient Anti–Migratory Target for Nanocapsules

**Figure 4:**
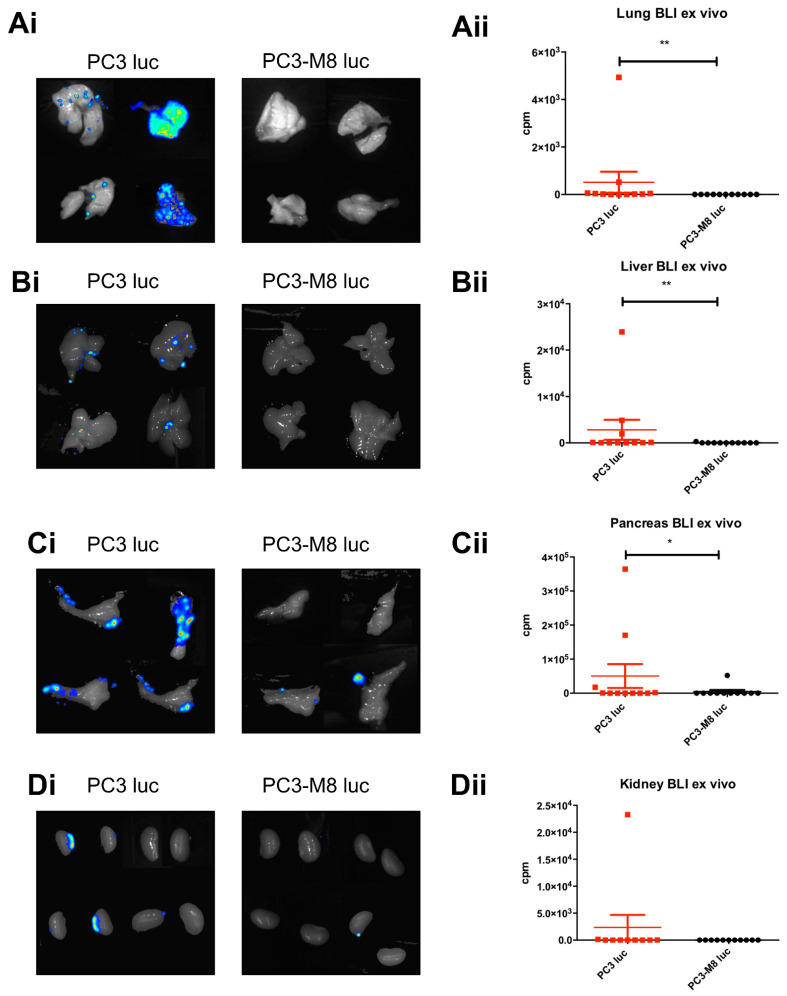
TRPM8 overexpression inhibits PCa cells’ metastatic dissemination. Representative images of bioluminescence in the lungs (**Ai**), liver (**Bi**), pancreas (**Ci**), and kidneys (**Di**) of mice 5 weeks after orthotopic grafts with PC3 luc (red dots) or PC3–M8 luc (black dots). Quantification of bioluminescence in the lungs (**Aii**), liver (**Bii**), pancreas (**Cii**), and kidneys (**Dii**) of mice 5 weeks after orthotopic grafts (n = 11 mice/condition; statistical significance: * = *p* < 0.05; ** = *p* < 0.01, Mann–Whitney test).

To further characterize the role of TRPM8 in PCa progression, the metastatic dissemination of PC3 luc and PC3–M8 luc from orthotopic grafts shown in Figure 1 was analyzed by bioluminescence imaging. As shown in Figure 4, we found a higher metastatic rate in mice grafts with PC3 luc compared with those grafted with TRPM8–overexpressing PC3–M8 luc cancer cells (Figure 4). In particular, we observed metastases in different sites, such as the lungs, liver, pancreas, and kidneys, which were significantly less abundant in PC3–M8 luc xenografts (Figure 4A–D). Nevertheless, on the basis of these in vivo experiments, we cannot conclude whether TRPM8 overexpression also acts on metastasis dissemination because of the large difference in tumor growth between the PC3 luc and PC3–M8 luc orthotopic grafts.

**Figure 5:**
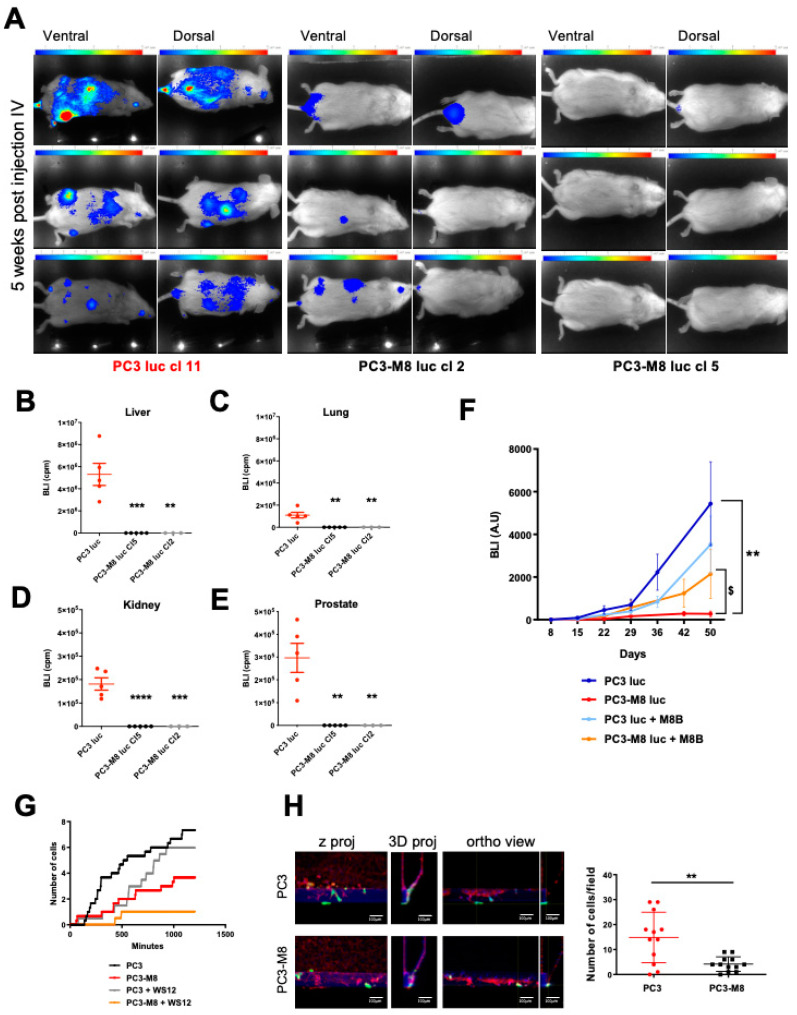
TRPM8 overexpression in PC3 inhibits metastatic dissemination. PC3 luc or PC3–M8 luc cells were engrafted into NSG mice by tail vein injection, and tumor burden was noninvasively monitored by measuring the bioluminescence signal generated upon subcutaneous administration of luciferin. (**A**) Representative bioluminescence images of PC3 luc and PC3–M8 luc in mice 5 weeks after intravenous injection into the tail vein (n = 5 mice/condition). (**B**–**E**) Quantification of bioluminescence signal in the liver (**B**), lungs (**C**), kidneys (**D**), and prostate (**E**) 5 weeks after PC3 luc (red dots); PC3–M8 luc cl5 (black dots) or PC3–M8 luc cl2 (grey dots) tail vein injection (n = 5 mice/condition; statistical significance: ** = *p* < 0.01, *** = *p* < 0.001, **** = *p* < 0.0001, ordinary one–way ANOVA with post hoc Tukey’s test). (**F**) Quantification of bioluminescence in mice after intracardiac injection of PC3 luc or PC3–M8 luc cells in the presence or absence of TRPM8 antagonist M8B (n = 5 mice/condition; statistical significance versus PC3 luc: ** = *p* < 0.01, statistical significance versus PC3–M8 luc: $ = *p* < 0.05, Student’s *t*–test). (**G**) Trans–endothelial assay of PC3 and PC3–M8 cells treated or not treated with WS12 (10 nM). Data are shown as the number of cells that migrated under the endothelial barriers over time (n = 5). (**H**) Immunofluorescent staining of microfluidic co–cultures performed on organ–on–chip systems (scale bar = 100 μm). Left and central panels: representative confocal images and 3D reconstruction of cells’ extravasation (GFP–tagged cells in green, vessels stained with phalloidin in red, and nuclei stained with DAPI in blue); right panel: quantification of PC3 (red dots) and PC3–M8 (black dots) extravasated into the matrix (n = 12; statistical significance: ** = *p* < 0.01, unpaired *t*–test).

Therefore, we injected PC3 luc and PC3–M8 luc into the tail veins of the mice to more clearly define the role played by TRPM8 in PCa cell dissemination and organ colonization in vivo. As shown in Figure 5A, PC3 luc cells markedly disseminated into the mice, resulting in the development of metastatic tumors 5 weeks after injection, while the injection of PC3–M8 luc cells did not induce any development of metastases (Figure 5A). More precisely, looking at the total mouse bioluminescence data (Supplementary Figure S1A), metastasis development was reduced by 97.32 ± 1.75% after PC3–M8 luc cl2 injection and by 99.45 ± 0.44% after PC3–M8 luc cl5 injection in comparison with control (PC3 luc) injection. As revealed by bioluminescence imaging performed in different organs after mouse sacrifice, PC3–M8 luc injection resulted in very restricted tumor colonization in the prostate, liver, kidneys, and lungs compared with PC3 luc injection (Figure 5B–E). To further validate TRPM8′s involvement in PCa cell dissemination in vivo, we injected PC3 luc and PC3–M8 luc intracardially into the left ventricles of NMRI nude mice. Mice were then injected intraperitoneally three times per week with the TRPM8 inhibitor M8B or its solvent for the control group. As shown in Figure 5F, we found a significant difference between mice grafted with PC3 luc and those grafted with PC3–M8 luc in terms of bioluminescent foci, indicating that a strong reduction in tumor cell dissemination was carried out by TRPM8 (Figure 5F). Furthermore, TRPM8 inhibition with M8B led to the partial recovery of PCa cells’ capability to migrate and disseminate through the body. Indeed, we found that mice injected with PC3–M8 luc and treated with M8B for 50 days displayed a significantly higher bioluminescence signal compared with their untreated littermates (Figure 5F), confirming once again the role TRPM8 plays in this process.

Next, to evaluate the possible involvement of TRPM8 in PCa blood intra–and/or extravasation, trans–endothelial migration assays were performed. As shown in Figure 5G, TRPM8 overexpression significantly decreased the ability of PCa cells to pass through a monolayer of endothelial cells by 33.85 ± 8.28% (statistical significance between PC3 and PC3–M8: *p*–value = 0.004). To further validate this finding, MIMETAS organ–on–chip technology was used, allowing us to compare PC3 and PC3–M8 cells’ behavior within HMEC 3D micro–vessels in a standardized microfluidic platform (Figure 5H). This assay demonstrated TRPM8′s capability to affect the trans–endothelial process, reducing PC3 extravasation by 71.90 ± 5.68%.

**Figure 6:**
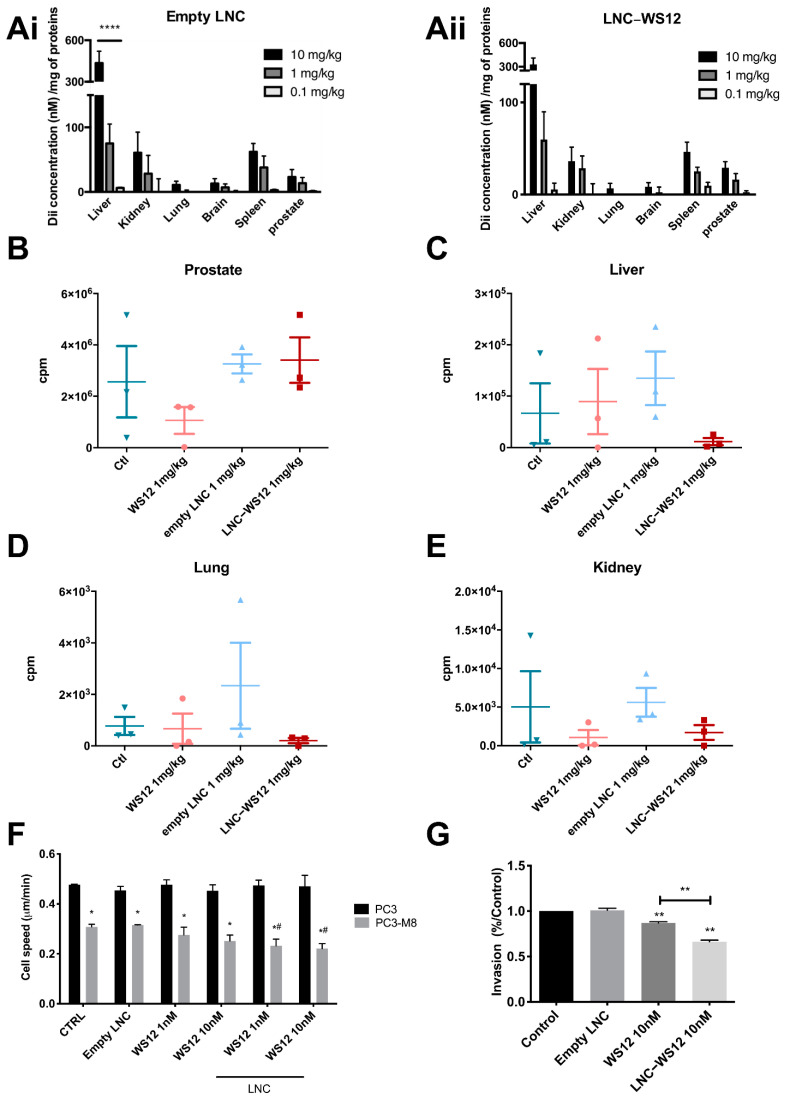
Effects of lipid nanocapsules (LNC) containing a TRPM8 agonist on migratory and invasive properties. (**A**) Biodistribution of empty LNC (**Ai**) and LNC WS–12 (**Aii**) labeled with DiI in mice. Mice were injected three times per week for 3 weeks with 10, 1, or 0.1 mg/kg LNC concentrations. Biodistribution was quantified using DiI concentration per mg of protein in each organ (n = 5 mice/condition; statistical significance: **** = *p* < 0.0001, two–way ANOVA with post hoc Tukey’s test). (**B**–**E**) Bioluminescence quantification in the prostate (**B**), liver (**C**), lung (**D**), and kidneys (**E**) of mice xenografts after PC3–M8 luc injection and treatment with free WS12, LNC–WS12s, or empty LNCs three times per week for 5 weeks (n = 3 mice/condition). (**F**) Random migration assay of PC3 cells stably expressing TRPM8, treated or not treated with free WS12 or LNC–WS12s (1 or 10 nM) (n = 3 independent experiments with, globally, at least 100 cells/condition; statistical significance between PC3 and PC3–M8 in each condition: * = *p* < 0.05; statistical significance between PC3–M8 not treated and PC3–M8 treated with 1 and 10 nM LNC–WS12 concentrations: # = *p* < 0.05, two–way ANOVA with post hoc Tukey’s test). (**G**) Invasion assay of PC3–M8 cells on Matrigel^®^ transwell (n = 3 independent experiments at least; statistical significance versus untreated PC3–M8 cells used as control: ** = *p* < 0.01, RM one–way ANOVA with post hoc Tukey’s test).

Once the anti–metastatic role played by TRPM8 in PCa dissemination was confirmed, we investigated the possibility of using TRPM8 as a therapeutic target in PCa treatment. To accomplish this, a formulation previously developed and characterized [21] containing the TRPM8 agonist WS12 incorporated into lipid nanocapsules (LNC–WS12s) was used. First, we evaluated the biocompatibility and biodistribution of LNCs in vivo after injecting them at three different concentrations (0.1, 1, and 10 mg/kg) into the tail veins of mice three times a week for 3 weeks (Figure 6A). Empty LNCs or LNCs containing WS12 were not toxic since they did not increase mortality in mice or change their behavior during the treatment. Concerning the LNC biodistribution, both empty LNCs (Figure 6(Ai)) and LNC–WS12s (Figure 6 (Aii)) displayed a preferential accumulation in the liver, spleen, and kidney, as well as in the prostate at the two highest doses (1 and 10 mg of LNC/kg but not with 0.1 mg/kg). Since the highest dose tested (10 mg/kg) showed a strong accumulation in the liver, and the lowest dose (0.1 mg/kg) was nearly undetectable in the prostate, we chose 1 mg/kg for further in vivo investigations in our prostate orthotopic xenografted mouse model. Three days following PC3–M8 luc injection, mice were treated three times per week for 5 weeks with empty LNCs or LNC–WS12s (1 mg/kg); mice treated with free WS12 or vehicle (CTRL) were used as controls. After sacrifice, organs were analyzed by bioluminescence in the primary prostatic tumor as well as at metastatic sites, such as the liver, lungs, and kidneys (Figure 6B–E). Regarding the primary tumor site, only free WS12 decreased PC3–M8 luc cells in the prostate, with a reduction of 58.64 ± 16.47% compared with the control condition, although no statistical significance was detected (compared with treatment with the vehicle, i.e., empty LNCs). On the other hand, in the liver and lungs, treatment with LNC–WS12 was the most efficient since it decreased the presence of PC3–M8 luc cells by 82.54 ± 17.45% and 73.56 ± 26.43%, respectively. Finally, concerning the kidneys, free WS12 and LNC–WS12 treatments displayed the same effect, with a decrease in PC3–M8 luc cells of 78.93 ± 21.06% in mice treated with free WS12 and 65.99 ± 34.01% in mice treated with LNC–WS12s. Overall, our results clearly show that the activation of TRPM8 by WS12, either free or encapsulated in LNCs, can effectively reduce the metastatic dissemination of PC3–M8 luc in mice, supporting the anti–metastatic role of the TRPM8 channel in PCa progression.

The results observed in vivo were further validated in vitro through random migration assays, which confirmed that TRPM8 overexpression significantly reduced the cell migration speed by 35.51 ± 1.06% (Figure 6F). Moreover, we showed that TRPM8 activation by both free WS12 and LNC–WS12s further decreased in vitro cell migration, while no effect was observed in PC3 luc cells lacking TRPM8 expression (Figure 6F). Interestingly, the encapsulation of WS12 into LNCs resulted in an additional 10% reduction in cell migration. Besides migration, cancer cells need to cross the extracellular matrix to reach the bloodstream and colonize a new site other than the primary tumor. To study the effect of TRPM8 on PC3 cell invasion, Matrigel–coated transwell assays were performed (Figure 6G). The treatment of PC3–M8 luc cells with LNC–WS12s reduced cell invasiveness more potently than treatment with free WS12, and this effect was even more pronounced than that in simple migration assays. Indeed, the activation of TRPM8 by WS12 in its free form reduced the invasiveness of PC3–M8 cells by 13.06 ± 1.32%, while the stimulation of TRPM8 by WS12 encapsulated in LNCs reduced it by 32.72 ± 1.83%. No effect on invasion was observed in PC3 luc cells (Supplementary Figure S1B).

### 2.4. TRPM8 Activation Inhibits Different Signaling Pathways, Leading to a Global Reduction in Tumor Growth and Metastasis Dissemination

**Figure 7:**
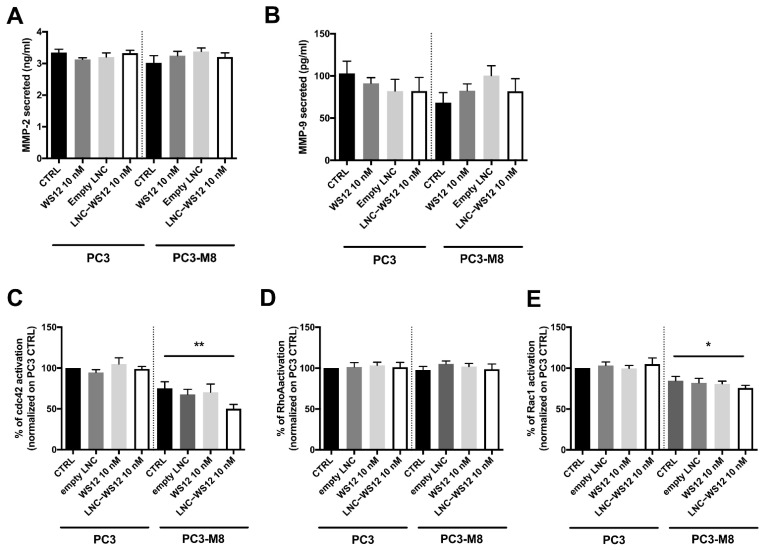
TRPM8 does not affect MMP secretion but modulates Rho GTPase signaling pathway. (**A**,**B**) MMP–9 (**A**) and MMP–2 (**B**) secretion in PC3 luc and PC3–M8 luc treated with WS12 (10 nM), empty LNCs, or LNC–WS12s (10 nM); data are expressed as means ± SEM of six independent experiments (statistical significance: ordinary one–way ANOVA with post hoc Tukey’s test); (**C**–**E**) Activation of Cdc42 (**C**), Rac1 (**D**), and RhoA (**E**) in PC3 luc and PC3–M8 luc treated with WS12 (10 nM), empty LNCs, or LNC–WS12s (10 nM). Data are normalized to the CTRL (PC3 luc) and expressed as means ± SEM of five independent experiments for each condition (statistical significance: * = *p* <0.05, ** = *p* <0.01, ordinary one–way ANOVA with post hoc Tukey’s test).

To better elucidate the intracellular pathways underlying the inhibitory effect of TRPM8 on PCa growth and metastasis dissemination observed both in vitro and in vivo, we evaluated different signaling pathways. First, we evaluated the possible impact of TRPM8 overexpression and/or activation on the secretion of matrix metalloproteinases (MMPs), such as gelatinases MMP–2 and –9, which are well–known to promote cell migration and invasion. However, MMP–2 and MMP–9 secretion was not affected by TRPM8 overexpression or activation (Figure 7A,B). Next, we investigated the involvement of small Rho GTPases because of their well–established role in the regulation of cell migration [22]. The activation of small Rho GTPases, including Cdc42, Rac1, and RhoA, upon TRPM8 overexpression and/or activation was investigated in vitro and showed that TRPM8 induced a significant decrease in GTP–bound (active) Cdc42 (Figure 7C) and Rac1 (Figure 7D) activation, but it did not affect RhoA activity (Figure 7E). More precisely, TRPM8 overexpression reduced Cdc42 and Rac1 activity by 24.64 ± 7.91% and 15.26 ± 5.17%, respectively, but that inhibition became significant only after TRPM8 activation by LNC–WS12s (Figure 7C,D).

**Figure 8:**
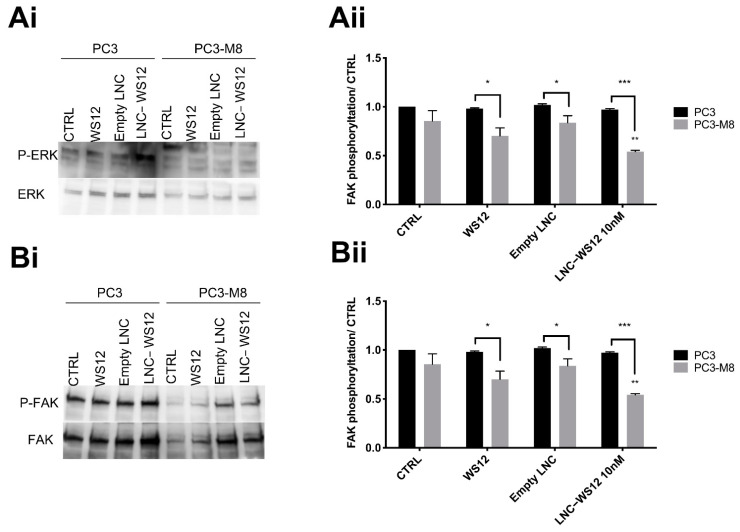
TRPM8 affects ERK and FAK phosphorylation, as assessed by Western blot. Representative blots showing ERK (**Ai**) and FAK (**Bi**) phosphorylation in PC3 luc and PC3–M8 luc cells treated with WS12 (10 nM), empty LNCs, and LNC–WS12s (10 nM); quantification of ERK (**Aii**) and FAK (**Bii**) phosphorylation normalized to the control condition (n = 3 independent assays, * = *p* < 0.05; ** = *p* < 0.005; *** = *p* < 0.001, two–way ANOVA with post hoc Tukey’s test).

Finally, we checked whether TRPM8 overexpression and/or activation could affect cell migration through the regulation of focal adhesion. For this purpose, we investigated whether TRPM8 had an impact on extracellular signal–regulated kinases (ERK) and focal adhesion kinase (FAK) phosphorylation (Figure 8A,B, respectively). ERK1/2 and FAK phosphorylation was evaluated by Western blot and indicated that TRPM8 overexpression and activation by free WS12 (10 nM) were not sufficient to regulate ERK and FAK activation (Figure 8). However, TRPM8 activation by LNC–WS12s (10 nM) induced a significant decrease in FAK phosphorylation by 36.4 ± 2.6% and 26.9 ± 2.5%, respectively (Figure 8(Aii,Bii)).

## 3. Discussion

A central role in the regulation of PCa cell progression has been previously ascribed to TRPM8, making it one of the most promising clinical targets for PCa therapy. Currently, TRPM8 is already used as a clinical diagnostic/prognostic marker in some countries [11,12]. Indeed, TRPM8 channel expression changes during PCa progression, revealing an upregulation in the early stages of both benign prostate hyperplasia and malignant prostate carcinoma followed by downregulation and silencing during late metastatic stages of PCa after hormonal therapy against androgens [23,24,25]. This is mainly due to the genomic and non–genomic androgen–dependence of TRPM8 [26,27]; during anti–androgen therapy, some cells develop the ability to escape this treatment by acquiring a more aggressive androgen–independent phenotype with the subsequent loss of TRPM8 expression/activity. Consistent with this expression profile, several studies have suggested a protective role of TRPM8 in metastatic PCa progression because of its inhibitory impact on PCa cell proliferation, viability, and migration [28]. However, some data reported in the literature are contradictory, and furthermore, most of them are based on in vitro studies. In this work, we clarified the role played by TRPM8 in PCa progression in vivo, mainly focusing on its effects on tumor growth and metastasis dissemination.

TRPM8 had an effect on cell proliferation in prostate tumor cells but not in normal prostate cells [20]. However, the role of TRPM8 in the regulation of PCa proliferation and apoptosis has been assessed by different groups using several in vitro experiments, which have led to contradictory conclusions. In particular, data from the literature have reported opposite effects of TRPM8 on the proliferation of different PCa cell lines according to their androgen sensitivity. More specifically, TRPM8 was found to be essential for cell survival and proliferation in PCa cells sensitive to androgens, such as LNCaP cells, in vitro [16,20,29], whereas it displayed an anti–proliferative and pro–apoptotic effect on androgen–insensitive PCa cells, such as PC3 and DU–145 cells [13,14,15]. This androgen receptor (AR)–dependency of TRPM8′s role in proliferation has also been observed in cells from other cancers, such as colon, osteosarcoma, and lung cancer [30,31,32]. In this context, our in vivo investigations support the anti–proliferative role of TRPM8 in PC3 cells, in line with the only other in vivo study on TRPM8 reported in the literature by Zhu and colleagues [14]. Indeed, we found that in mice grafted with luminescent PC3 cells overexpressing TRPM8, prostate tumor growth dramatically decreased starting 4 weeks after the orthotopic graft. TRPM8 expression not only reduced tumor weight and area, but also affected its histological features, helping to maintain well–defined glandular tissue. Notably, to date, no data were available on the tumorigenesis of PCa cells grafted in the prostate. Mechanistically, our in vitro data revealed that the inhibition of tumor growth observed in vivo was accompanied by a reduction in the proliferative cell fraction and increased cell cycle arrest in the G_0_/G_1_ phase induced by TRPM8 overexpression. These findings are in agreement with previous data showing that TRPM8 activation by menthol in DU145 cells [15] and TRPM8 overexpression in PC3 cells [13] induced G_0_/G_1_ cell cycle arrest, downregulating cyclin–dependent kinase (Cdk) 4 and Cdk6 [13]. Moreover, a significant decrease in proliferating cell nuclear antigen (PCNA) in the presence of TRPM8 was detected in vivo [14]. Nevertheless, conflicting results showed that TRPM8 pharmacological inhibition by BCTC impaired DU145 cell cycle progression, downregulating key proteins, including protein kinase B, cyclin D1, Cdk2, and Cdk6, and upregulating others, such as glycogen synthase kinase 3β [33]. Moreover, MAPK pathways also seem to be involved in the aforementioned pro–proliferative action of TRPM8 in DU145 cells [33]. In this contradictory landscape, our in vitro and in vivo data further support the anti–proliferative role of TRPM8 in PCa progression. In addition, we observed a concomitant reduction in the apoptotic and necrotic areas of ex vivo PC3–M8 tumors compared with PC3 tumors. However, no differences were observed in vitro in terms of apoptosis between PC3 and TRPM8–overexpressing PC3 cells. Therefore, we can conclude that TRPM8 is not directly involved in the control of cell apoptosis and that the difference observed in the apoptotic fraction ex vivo is instead the result of the normal tumor growth of PC3 with a necrotic area at the center and an apoptotic area next to it. Conversely, TRPM8 inhibition or suppression in LNCaP cells led to the induction of pro–apoptotic pathways, supporting a TRPM8–mediated anti–apoptotic mechanism in androgen–sensitive PCa cells [16]. This role may be explained by the androgen–dependent expression of TRPM8 in the ER of LNCaPs, in which TRPM8 was shown to induce plasma membrane (PM) store–operated channel (SOC) currents [29] sufficient to induce the apoptotic process [34]. In this context, it was also demonstrated that LNCaP cells express a short (19 kDa) TRPM8 isoform, sM8α, which is able to negatively regulate TRPM8 full–length channels by interaction [35], thus inhibiting apoptosis [36].

Beyond cell proliferation and apoptosis, another cell hallmark that may affect tumor growth is the clonogenic ability of cells. This process seemed to contribute the most to the marked differences observed in vivo between tumors from the PC3 and PC3–TRPM8 orthotopic grafts. Indeed, we showed that the overexpression and especially the activation of TRPM8 significantly inhibited PCa cells’ clone formation capabilities. This finding was further supported by the decreased tumor cell density observed ex vivo in PC3–TRPM8 tumors compared with PC3 tumors. The inhibition of Cdc42 and Rac1 activity we observed after TRPM8 overexpression and/or activation could account for such a decrease. Indeed, the negative dominant expression of Rac1 or Cdc42 (Rac1–N17 or Cdc42–N17) in kidney cells (MDK cells) was shown to decrease cell–to–cell adhesion, explaining the reduced clonogenic ability and tumor cell density [37,38]. Similarly, the active mutant of Rac1 (Rac1–V12) increases paracellular permeability and expression of E–cadherin to promote cell–to–cell adhesion [38,39]. Taken together, the effects of TRPM8 on clonogenic cell ability and, to a lesser extent, cell proliferation may explain the inhibition of prostate tumor growth observed in vivo. However, the tumor microenvironment must also be taken into account in these kinds of considerations since it could affect tumor growth via paracrine signaling [40]. The tumor microenvironment is composed of immune cells and fibroblasts, which can be modulated by tumor cells and transformed into cancer–associated fibroblasts (CAF). Through this transformation, CAF may secret VEGF or interleukin–6 (IL–6), thus affecting angiogenesis and tumor growth [41]. The cellular context could also be involved in the definition of TRPM8′s role in cancer progression. Interestingly, an intriguing association between TRPM8 overexpression and a strong in vivo reduction in VEGF and microvascular density (MVD) has been reported [14], thus highlighting a possible additional TRPM8–mediated, anti–angiogenic contribution to the observed reduction in tumor growth in vivo. Therefore, TRPM8 could be considered a useful target to block PCa growth by using TRPM8 antagonists, such as capsazepine [16], BCTC [20], cannabigerol [42], as well as TRPM8 agonists, including menthol [15,16] and D–3263 [43], depending on the cancer stage. Nanocarriers loaded with TRPM8 agonists, such as WS–12, could thus be used in early cancer stages when TRPM8 is highly expressed in order to avoid tumor cell dissemination.

High cancer–related mortality is essentially due to tumor metastasis. This process mainly relies on cancer cell migration followed by intra– and extravasation into the blood and lymphatic circulation. In this regard, TRPM8 has already been studied and characterized, outlining a protective anti–migratory role of TRPM8 in vitro [13,15,17,18,21,27,44]. Here, using orthotopic prostate xenografts and lateral caudal vein injection of PC3 and PC3–TRPM8 into immunocompromised mice, we further demonstrated that TRPM8 exerts an inhibitory action on PCa cell migration in vivo. Indeed, we showed that TRPM8 overexpression reduced PC3 cell colonization in primary and distant sites, such as the liver, kidneys, and lungs. This outcome is at least partially attributable to the inability of PC3 overexpressing TRPM8 to disseminate to different metastatic sites through the bloodstream by overcoming the vascular barrier. Indeed, in vitro trans–endothelial migration assays demonstrated that TRPM8 overexpression strongly inhibited intra– or extravasation of PC3 cells through a monolayer of endothelial cells. This lack of intra– or extravasation due to TRPM8 overexpression could be explained by the TRPM8–induced inhibition of cell adhesion, which we previously described in endothelial cells [18], confirming what was previously observed in PCa epithelial cells [14]. Indeed, in endothelial cells, the TRPM8–mediated inhibition of cell migration occurs through the direct interaction of the channel with the small GTPase Rap1A and the consequent inhibition of the integrin inside–out pathway, blocking cell adhesion and migration [18]. The TRPM8–mediated inhibition of cell adhesion could also partially explain the decrease we observed in the clonogenic ability of PC3 cells overexpressing TRPM8. TRPM8 overexpression by itself inhibits PCa cell migration, and TRPM8 activation by endogenous and exogenous agonists, such as prostate–specific antigen (PSA), icilin, menthol, and WS12, further enhances the inhibitory effect of metastatic PCa cell motility [13,14,17,21].

During metastatic dissemination, cancer cells secrete matrix metalloproteinases (MMPs) to degrade the extracellular matrix, facilitating their migration and invasion. Mechanistically, TRPM8 does not affect the invasion process since neither TRPM8 overexpression nor activation affected MMP–2 and MMP–9 secretion in PC3 cells. However, TRPM8 played an active role in another key aspect of cell migration—the capability of cells to create focal adhesion to promote cell movement. This process is regulated by several proteins, including some small GTPase belonging to the Rho superfamily, such as Cdc42, Rac1, and RhoA. These proteins are located at the front of the cell and are responsible for the formation of protrusions and focal adhesions essential for cell adhesion and migration [22]. Interestingly, we found that TRPM8 activation reduced Cdc42 and Rac1 activity but not RhoA activity. Taking into account the roles played by Cdc42, Rac1 protein, and their effectors in the epithelial–to–mesenchymal transition (EMT) through cytoskeleton remodeling [45], the TRPM8–mediated inhibition on cell invasiveness we observed might be explained as a decrease in the EMT through Rac1 and Cdc42 inhibition. Moreover, we showed that TRPM8 activation also inhibited the phosphorylation of two important kinases involved in focal adhesion formation, i.e., ERK and FAK. These findings are in line with other studies that have reported a TRPM8–mediated inhibition of DU145 [15] and PC3 cell motility supported by the downregulation of phospho–FAK without changing non–phospho–FAK [13,14]. In addition, we recently demonstrated that the inactivation of TRPM8 by low doses (10 nM) of testosterone significantly increased FAK phosphorylation, consequently promoting cell migration [27]. Interestingly, newly identified partner proteins of channels, such as PSA, TRP channel–associated factors 1 (TCAF1), and AR, were shown to affect the expression of TRPM8 on the plasma membrane, its opening state, and thus the TRPM8–mediated inhibitory effect on cell migration [17,44,46]. Consistently, the short TRPM8 isoform sM8α expressed by LNCaP cells promoted cell migration, acting as an inhibitory partner protein of full–length TRPM8 [36]. Nevertheless, a contrasting study showed that the pharmacological inhibition of TRPM8 by BCTC may reduce the speed of PCa DU145 cells [33]. However, most lines of evidence, including the present study, strongly suggest an anti–migratory role of TRPM8, shedding light on the possibility of using TRPM8 agonists, such as PSA, WS12, and icilin, to counteract the metastasis of prostate cancer [17,21].

Since TRPM8 activation reduces the migratory PCa cell potential further than the simple overexpression of the channel, we aimed to develop a molecular tool to target TRPM8 for PCa treatment. In this context, we recently described the synthesis and the functional characterization of lipid nanocapsules (LNCs) containing the TRPM8 agonist WS12 [21]. Nanocarriers were developed and used in several forms, such as liposomes, virosomes, solid lipid nanoparticles, polymeric nanoparticles, and protein conjugates, to improve drug bioavailability and targeting in cancer therapy [47]. For example, nanoparticles containing the TRPA1 activator curcumin efficiently targeted PCa growth [48]. The LNCs we developed were composed of an oily liquid core containing our compound of interest and surrounded by a layer of lecithin and hydrophilic surfactants, which gave them a hybrid structure between polymeric nanocapsules and liposomes (lipoprotein–like structure) [49]. We showed that WS12 encapsulation in LNCs potentiated TRPM8 activation and, subsequently, the TRPM8–mediated inhibition of PCa cell migration in vitro [21]. Indeed, the use of nanocarriers not only allowed the use of an agonist concentration 10 times lower than that of free agonists to activate TRPM8, but it also ensured more efficient cellular delivery by overcoming the problem associated with the hydrophobicity of most activators of TRPM8. Here, we further supported the applicability of this therapeutic approach in vivo by injecting LNC–WS12s into our prostate orthotopic xenografted mouse model. We demonstrated that LNC–WS12s efficiently blocked PCa cell dissemination, particularly in the liver and lungs, where LNCs accumulated after lateral caudal vein tail injection. However, this tool seems to not be very efficient for targeting primary tumors in the prostate because of the low distribution of LNCs in the prostate when injected at 1 mg/kg. Nevertheless, because of their distribution in metastatic sites (lung or liver), LNC–WS12s could be used to target metastasis dissemination in patients with prostate cancer before anti–androgenic therapy and the subsequent loss of TRPM8 expression [23]. As we demonstrated that TRPM8 activation inhibited PCa cell trans–endothelial migration, LNC–WS12s could also represent an efficient strategy to target the extravasation of circulating tumor cells if TRPM8 expression is maintained in these cells. Moreover, as mentioned above, since some studies suggest a correlation between TRPM8′s role in prostate cancer progression and the androgen–dependent state of PCa cells, the efficacy of LNC–WS12s needs to be tested on androgen–dependent PCa cells.

Overall, this study reinforces the hypothesis that activating TRPM8 could play a protective role in prostate cancer progression, thus supporting its potential application as a powerful therapeutic target antagonizing the metastatic transition of PCa.

## 4. Materials and Methods

### 4.1. Cell Culture

Human prostate cancer cells from bone metastases (PC3, ATCC^®^) with stable luciferase overexpression (PC3 luc) and PC3 cells with TRPM8 and luciferase overexpression (PC3–M8 luc) were used in this study. Cells were obtained by stable transfection with pcdna4–TRPM8 and/or pcdna3 luciferase vectors. In order to obtain stable clones, transfected cells were selected using 100 µg/mL zeocin (InvivoGen) and/or 700 µg/mL geneticin (Sigma–Aldrich, Saint–Quentin Fallavier, France) every two passages. PC3 cells were grown in RPMI (Invitrogen) supplemented with 10% of fetal bovine serum (Pan Biotech, Aidenbach, Germany), L–glutamine (5 mM; Sigma–Aldrich), and PenStrep^®^ (100 mg/mL; Sigma–Aldrich).

The human microvascular endothelial cell line (HMEC–1, ATCC^®^) was cultured in EndoGRO™ MV–VEGF complete medium (Merck Millipore) supplemented with L–glutamine (5 mM; Sigma–Aldrich) and PenStrep^®^ (100 mg/mL; Sigma–Aldrich).

### 4.2. Lipid Nanocapsule Formulation

Lipid nanocapsules (LNCs) were formulated with a mixture of LabrafacTM Lipophile WL 1349 (caprylic/capric triglyceride), Phospholipon^®^ 90G (soybean lecithin at 97.1% of phosphatidylcholine), and Solutol^®^ HS15 (a mixture of free polyethylene glycol 660 and polyethylene glycol 660 hydroxystearate) generously provided by Gattefosse SAS (Saint–Priest, France), Phospholipid GmbH (Köln, Germany), and Laserson (Etampes, France), respectively. Type I ultrapure water was obtained from a Milli–Q plus system (Millipore, Paris, France). WS–12 was obtained from Tocris (Bio–Techne SAS, Noyal Châtillon sur Seiche, France). Other chemical reagents were obtained from Sigma–Aldrich (Saint–Quentin Fallavier, France), and solvents were obtained from Thermo Fischer Scientific (Illkirch, France) and used as received.

The formulation of LNC–WS12s for in vivo experiments was as follows. LNCs with a size of 25 nm were prepared with slight modifications in water using a phase inversion method of an oil/water system, as described by Heurtault et al. [11]. Typically, the oil phase containing Labrafac (2.52 g), Solutol (4.08 g), and Phospholipon 90G (0.375 g) was mixed with the appropriate amounts of WS–12 (29 mg, 1% of Labrafac + Phospholipon 90 G), Milli–Q water (5.4 mL), and NaCl (660 mg) and heated, under magnetic stirring, to 70 °C. The mixture was subjected to 3 temperature cycles from 26 to 76 °C under magnetic stirring. The mixture was then cooled to 45 °C before the addition of 28.2 mL of cold ultrapure water (0 °C). The formulation was stirred at room temperature for another 10 min and was stored in the fridge at 4 °C overnight before purification. Empty LNCs were formulated without WS12. The LNC suspensions were purified by dialysis (12,000–14,000 Da, 4 times) for 2 days in NaCl solution (9 g/L). The purified LNC suspensions were sterilized using syringe filters (0.22 µm) in a sterile plastic tube.

The drug loading of purified LNC suspensions was quantified by high–performance liquid chromatography (HPLC). High–performance liquid chromatography (HPLC) analysis was carried out on a Shimadzu LC2010–HT (Shimadzu, Tokyo, Japan) using a 5 µm C18AQ Uptisphere^®^ X–serie 300 Å, 250 × 4.6 mm column (Interchim, Montluçon, France) heated to 30 °C. The mobile phase consisted of a mixture of eluent A (formic acid 0.1% in H2O) and eluent B (formic acid 0.1% in acetonitrile) at a flow rate of 1 mL/min. The isocratic flow (eluent 1) was carried out for 1 min, and the linear gradient was 0 to 80% of eluent B for 10 min and 80% of eluent B for 4 min. The detection was performed at 254 nm. Diluted LNC solutions (by 10) were analyzed by injecting 40 µL into the column. A stock solution of WS–12 was prepared at 1 mg/mL in DMSO for the calibration curve. Concentrations of 5, 10, 20, 40, 80, and 120 µg/mL of WS–12 in DMSO were prepared from this stock and injected (40 µL) into the column. A calibration curve (Y = 127869*X + 40563, r² = 0.9999, LOD = 1.36 µg/mL, LOQ = 4.13 µg/mL) was obtained by linear regression of the drug concentration (X, µg/mL) versus the peak area (Y).

### 4.3. In Vivo Tumor Models

Ten–week–old male NMRI nude mice (Charles River Laboratories) were injected in the prostate with 2 × 10^6^ PC3 luc or PC3–M8 luc cells (12 mice/condition) suspended in 30 μL PBS while under isoflurane anesthesia. Animal weight was measured every week for 5 weeks, and tumor growth was monitored using bioluminescence (BLI) measurement. To measure BLI, mice were injected with d luciferin intraperitoneally (150 mg/kg) 10 min before bioluminescence imaging (Φ imagerTM; BIOSPACE Lab). Photons emitted by cancer cells were counted and expressed in counts per minute (c.p.m.). At necropsy after 5 weeks, ex vivo BLI measurement for each collected organ was performed using d luciferin intraperitoneal injection (150 mg/kg), and the results were expressed as counts per minute (c.p.m.).

Unanesthetized twelve–week–old male NSG Mice (Charles River Laboratories) were placed into a plastic restraining device and injected with 5 × 10^6^ PC3 luc or PC3–TRPM8 luc cells suspended in 150 μL PBS (15 mice were injected with PC3 luc cells, 9 mice with PC3–M8 luc clone 5 and 6 mice with PC3–M8 luc clone 2 cells). Cells were injected into the lateral tail vein through a 25–gauge needle, as previously described [50]. Tumor growth was measured every week for 5 weeks using d luciferin intraperitoneal injection (150 mg/kg) 10 min before bioluminescence imaging, and the results were expressed as counts per minute (c.p.m). At necropsy, ex vivo BLI measurement for each collected organ was performed, as described above.

To test the toxicity of the lipid nanocapsules (LNCs), fourteen–week–old male NMRI nude mice (Charles River Laboratories) grafted orthotopically with 2 × 10^6^ PC3–M8 luc cells (3 mice/condition) were injected in the lateral caudal vein with empty or WS–12–loaded LNCs labeled with DiI at three different concentrations (10, 1, or 0.1 mg/kg diluted in NaCl), free WS–12, or the solvent (NaCl) for the control three times per week for three weeks. After 3 weeks, the mice were sacrificed, and the presence of LNCs in the organs was analyzed using DiI imaging on lysates.

Intracardiac manipulations were performed on 52 six–week–old male NMRI nude mice (Charles River Laboratories) bred and housed at the “Small Animal Imaging Center” of the TAAM Unit at the CNRS (Orléans, France) according to referral n°1166 from CECCO n°3 and APAFIS authorization #19911. The mice were anesthetized with an air/2% isoflurane mixture (Piramal, CSP) and then injected in the left ventricle with 2 × 10^6^ PC3 luc clone 11 or PC3–TRPM8 luc clone 2 cells suspended in 100 μL PBS. Starting from D1, the mice received 200 μL of a solution containing 2.7% DMSO (control group) or 200 μL containing 0.130 mg of the TRPM8 inhibitor M8B hydrochloride (Bio–Techne, Noyal Châtillon sur Seiche, France) diluted in 1X PBS containing 2.7% of DMSO (treated group). Treatments were administered intraperitoneally 3 times a week until D50. To assess the quality of the injection of cells into the left ventricle and then perform imaging of tumor proliferation, bioluminescence imaging was performed with an Ivis^®^ Lumina device (Perkin Elmer, Villebon–sur–Yvette, France)). Anesthetized mice were imaged by bioluminescence on both the ventral and the dorsal side once a week for 8 weeks upon injection of luciferin (Perkin Elmer, France, supplied by CIPA, Orléans, France)) at a dose of 2 mg/mouse.

### 4.4. Histology, Immunostaining, and Morphometric Analyses

Mouse tissue samples were fixed in 4% PFA overnight at 4 °C, dehydrated, and embedded in paraffin (for 8 μm serial sections). Histology was performed by Trichrome staining (histology core facility of Lille). Before immunofluorescence experiments, the sections underwent deparaffinization: the sections were incubated in xylene 2 times for 10 min, rinsed with 100% ethanol (2 times × 3 min), rehydrated in a decreasing gradient of Ethanol/H20 (100%, 96%, 70%, and 30%, 5 min each), and finally transferred to PBS. The sections were blocked for 30 min in blocking buffer (PBS + 10% Donkey serum + 0.3% triton X100) and then incubated with primary antibodies in blocking buffer overnight at 4 °C. After three rinses in PBS, the sections were incubated for 90 min at RT with secondary antibodies diluted in blocking buffer. Finally, the sections were rinsed three times in PBS before nuclear staining with DAPI (1:200 in PBS) and mounting with Mowiol. Immunostaining was performed using the following primary antibodies: Rb anti–Ki67 (Ab 15580, 1:100) and goat anti–TRPM8 (Antibodies ONLINE, 1:100)

Sections were then incubated with the appropriate fluorescently conjugated secondary antibodies (Dk anti–Gt Alexa 488 1:400 Molecular Probes, Dk and Rb Rodhamine 1:250 Jackson Immunoresearch). Nuclei were counterstained with DAPI (Invitrogen, Life Technologies, Ghent, Belgium). For morphometric analyses, mosaic tile images were taken by an Axio Scan, Z1 (Zeiss, Jena, Germany) with a ×20 dry objective (NA 0.8). Images were processed with ZEN software (Zeiss Efficient Navigation) and analyzed using the NIH ImageJ software by sequential operations, allowing for tumor area identification, necrotic area identification, and cell counting. For each necrotic area, the number of total cells (stained with DAPI) was counted using a fixed threshold and watershed, and particles were analyzed from 30 to 5000 pixels. Tumor proliferation was defined as the Ki67+ area and expressed as a percentage of the total nuclei (DAPI+). Apoptosis was measured by TUNEL following the manufacturer’s instructions (DeadEnd™ Fluorometric TUNEL System). Ki67 + cells or apoptotic cells were counted as GFP channels using a fixed threshold and watershed, and particles were analyzed from 30 to 5000 pixels. The complete macro is reported in the Supplementary Material as Github.

Confocal imaging was performed using an LSM700 confocal microscope (Carl Zeiss, Munich, Germany).

### 4.5. Flow Cytometry Assay

Cells were harvested, washed with PBS, and fixed with cold 70% ethanol for 30 min. After rinsing with PBS, cells were incubated with an RNase cocktail (Invitrogen) in PBS for 15 min, followed by 50 µg/mL propidium iodide (PI; Sigma Aldrich) for 30 min at room temperature. After incubation, cells were placed on ice before being analyzed by flow cytometry. Data were acquired with a CyAn ADP flow cytometer (Beckman Coulter, USA) using an FL3 channel (488 nm excitation, and 640DLP–613/20 filters). Analysis was performed using Summit software.

### 4.6. Apoptosis Assay

Tumor apoptotic cell death was detected and quantified via the DeadEndTM Fluorometric TUNEL System Kit (Promega) according to the manufacturer’s instructions. For apoptosis analysis of tumor sections, mosaic tile images covering an entire tumor section were acquired, and the TUNEL+ area was expressed as a percentage of the total tumor area analyzed.

### 4.7. Clonogenic Assay

The ability of the cells to form clones was assessed using a clonogenicity test. PC3 luc and PC3–M8 luc cells were cultured in a 6–well plate at a confluence of 800 cells/well for 2 weeks. The medium, which contained different treatment conditions (WS12 at 1 and 10 nM, empty LNCs, WS12–loaded LNCs at 1 and 10 nM, icilin at 10 µM, and M8B at 1 µM), was changed every three days during the time of the experiment. After 2 weeks of incubation, the clones obtained were rinsed with PBS, fixed with methanol, and stained with Crystal violet (Santa Cruz, CA, USA). The number of clones present in each well was counted using ImageJ software [51], and each condition was normalized to the control condition.

### 4.8. Time–Lapse Video Microscopy

Cells were seeded at a low density and kept at 37 °C under 5% CO_2_ in an incubator chamber for time–lapse video recording (Okolab). Cell movements were monitored with an inverted microscope (Eclipse Ti–E; Nikon Corporation, Tokyo, Japan) using a 10X/0.25 NA Plan objective lens.

Images were acquired every 10 min for a time–lapse of 10 h with a CCD video camera using NIS–Element software (Nikon). Image stacks were analyzed with ImageJ software, and at least 100 cells/condition were manually tracked using the MtrackJ plugin. We excluded dividing cells as well as cells that exited the imaged field during the time–lapse acquisition period. The mean speed parameter was considered for the data analyses. At least 6 fields for each condition were analyzed in each independent experiment. At least three independent experiments were performed for each experimental condition.

### 4.9. Invasion Assay

Cells were harvested from the culture dish, 5 × 10^4^ cells in 200 μL of 2% serum medium were transferred to Transwell inserts coated with Matrigel^®^ (the top compartment, 8–μm pore size, Corning, Sigma–Aldrich, Saint–Quentin Fallavier, France), and 1 mL of 10% serum medium was placed in the lower chamber. Following incubation at 37 °C for 22 h in a cell culture incubator, cells on the upper surface of the filters were removed with cotton swabs; the filters were washed with PBS, fixed in methanol, and stained with crystal violet. Cells that had moved to the lower surface of the filter were counted under the microscope. Migrated cells in each field were quantified. Results are presented as relative migration by setting the migrating cell number of control cells as 100.

### 4.10. Trans–Endothelial Migration Assay

To analyze the ability of PC3 and PC3–M8 cells to migrate through an endothelial cell monolayer, we used a video microscopic in vitro “extravasation” assay. In brief, a thin basement membrane–like matrix (consisting 1X RPMI, 10 mmol/L HEPES, 0.04 mg/mL laminin, 0.04 mg/mL fibronectin, and 0.32 mg/mL collagen IV, adjusted to pH 7.4) was layered onto a thick collagen I layer (containing 1X RPMI, 10 mmol/L HEPES, and 0.8 mg/mL collagen I, adjusted to pH 7.4 and incubated overnight at 37 °C in a 12.5 cm^2^ flask) for one hour at room temperature. Afterward, 1.7 × 10^6^ HMEC cells were added to the collagen double–layer and incubated for 7–10 days at 37 °C and 5% CO_2_. The medium was replaced every 2 days, and when the cells reached confluency, 10 ng/mL TNF–α were added. The next day, 3 × 10^5^ PC3 or PC3–M8 cells and 100 ng/mL epidermal growth factor (EGF) were added with or without senicapoc (30 μmol/L, dissolved in DMSO). Trans–endothelial migration was monitored for 13.5 h using a ZEISS microscope Axiovert 40C linked to a video camera (Hamamatsu, Germany). Images were taken in 5 min intervals. The analysis was performed using ImageJ. All PC3 and PC3–M8 cells within the given visual field were counted in the first image of the stack (t = 0). Then, individual cells were tracked. The criterion for trans–endothelial migration was that PC3 or PC3–M8 cells not only settle between but also clearly move underneath the endothelial cells. For these cells, the time point of intrusion into the endothelium was set to represent the beginning of trans–endothelial migration. For the final analysis, the number of transmigrated cells was normalized to the number of PC3 or PC3–M8 cells at t = 0. Three independent experiments were performed for each experimental condition.

### 4.11. Microfluidic Co–Cultures

For blood vessel formation, the OrganoPlate^®^ 2–lane (MIMETAS, 9603–400B) containing 96 chips (4.5 mm long gel and perfusion channels—200 μm × 120 μm (w × h)) was used to reproduce tubular endothelial structures that mimicked blood vessels in a microfluidic system [52]. In all experiments, rat tail collagen type I (Nalgene) was used as the extracellular matrix (ECM) for the cells (in the gel channel). A stock collagen type I solution (5 mg/mL) was neutralized with 10% NaHCO_3_ (37 g/L) and 10% HEPES buffer (1 M) (Sigma Aldrich) to obtain a final concentration of 4 mg/mL. NaHCO_3_ and HEPES were mixed at a 1:1 ratio (*v*/*v*), and then collagen I was added in a volume corresponding to 4 times the volume of the initial mix (final volume ratio 1:1:8 HEPES/ NaHCO_3_/Collagen I). Some attracting factors, such as FCS and VEGF, were added to the ECM. The neutralized collagen was kept on ice and used within 10 min. Next, 2 μL of the neutralized collagen was added into the inlet of each gel channel, and then the device was incubated for 30 min at 37 °C and 5% CO_2_ to allow collagen polymerization; the device was removed from the incubator and kept sterile at room temperature right before cell loading. HMEC cells were dissociated, pelleted, and resuspended in a complete medium (EndoGRO™ MV–VEGF, Merck Millipore) at a density of 2 × 10^7^ cells/mL. Next, 2 μL of the cell suspension was dispensed into the perfusion channel inlet followed by the addition of 50 μL of the culture medium to prevent dehydration of the cell suspension. The OrganoPlate^®^ was placed on its side with gel channels at the bottom and incubated (37 °C, 5% CO_2_) to allow the cells to adhere. After the cells were attached to the gel, the device was rotated back into an upright position, and 50 μL of the medium was added to the medium outlet. The device was then placed on a rocking platform (Perfusion rocker, MIMETAS) for continuous perfusion (7° inclination, 8 min cycle time). The culture medium was refreshed three times a week and right before each experiment.

For the extravasation assay, after transfection with TRPM8 and/or GFP plasmid, PC3 cells were inserted into the tubule (0.2 × 10^4^ cells/line), and the co–culture was imaged 16 h after PC3 injection using a Leica TCS SP5 confocal system (Leica Microsystems) with a magnification of 200×. DAPI (1:1000 for 15 min, Invitrogen, Life Technologies) was used for nuclear staining, and actin was labeled with Rhodamine–phalloidin (1:1000 for 15 min, Invitrogen, Life Technologies). The cells that passed through the tubule were counted, and they were divided into three zones: cells that had passed the tubule and were in the collagen I matrix, cells that were integrated into the tubule, and cells that rested in the tubule.

### 4.12. Western Blot Analysis

Cells were seeded in Petri dishes with the appropriate medium and grown to a confluency of 80%. Before cell lysis, Petri dishes were kept on ice and washed twice with ice–cold PBS. Cells were lysed with RIPA Buffer (25 mM Tris–HCl pH 7.6, 150 mM NaCl, 1% NP–40, 1% sodium deoxycholate, and 0.1% SDS) containing the following protease inhibitors: 2 mg/mL aprotinin, 1 mM Na orthovanadate, 0.1 mM PMSF, and 10 mM sodium fluoride. Lysates were centrifuged at 4 °C for 10 min at 12,000× *g*. Protein concentrations were determined using a Bicinchoninic Acid Kit (BCA kit, Sigma) following the manufacturer’s instructions. A quantity of 50 µg of the sample was resuspended in SDS sample buffer, heated at 95 °C for 5 min, and separated on 10% pre–cast SDS gel (Biorad). Polyvinylidene fluoride membranes were properly blocked and then incubated overnight with ERK1/2 (1:1000, #9102, Cell Signaling), phospho–ERK1/2 Thr402/Tyr204 (1:1000, #9101, Cell Signaling), AKT (1:3000, #9272, Cell Signaling), and Phospho–Akt–Ser473 (1:2000, #4508, Cell Signaling) antibodies. The membrane was then washed with TBS containing 0.1% Tween 20 and incubated with the appropriate horseradish–peroxidase–conjugated antibodies (Santa Cruz). Chemiluminescence assays were conducted using the SuperSignal West Dura chemiluminescent substrate (Thermo Fischer Scientific).

### 4.13. Cell Signaling Pathway Assays

Cell signaling activation pathways were analyzed using the Western blot technique to study the phosphorylation levels of ERK and FAK using the antibodies described above. To quantify the differences in protein phosphorylation, the ratio between non–phosphorylated and phosphorylated protein expression was evaluated using ImageJ software. Cdc42, Rac1, and RhoA activation was evaluated using a G–LISA^®^ activation assay kit for Cdc42, Rac1, and RhoA (G–LISA^®^ CDC42 Activation Assay Biochem KitTM, G–LISA^®^ Rac1 Activation Assay Biochem KitTM, G–LISA^®^ RhoA Activation Assay Biochem KitTM; Cytoskeleton). The manufacturer’s instructions were followed for each kit.

### 4.14. Statistical Analysis

Data are expressed as means ± SEM. The statistical significance of differences between groups was determined by analysis of variance (ANOVA) followed by pairwise comparison using Tukey’s or Bonferroni’s method for in vivo assays, flow cytometry, apoptosis assays, clonogenic assay, invasion assays, migration assay, and cell–signaling pathway assays. Student’s *t*–test was used to analyze the trans–endothelial migration assay. Differences with *p* < 0.05 were considered statistically significant. Statistical analyses were performed using GraphPad Prism 6 software (GraphPad Software Inc., San Diego, CA, USA)).

## 5. Conclusions

Our in vitro and in vivo results shed light on the role of TRPM8 on PCa AR–negative tumor growth and metastasis dissemination. In particular, we showed that TRPM8 overexpression decreased the proliferation and clonogenic properties of PCa cells, resulting in dramatically limited tumor growth in orthotopic graft mice. In addition to its role in prostate tumor growth, TRPM8 was shown to inhibit PCa cell migration and invasion, leading to reduced prostate metastasis dissemination in mice. Mechanistically, we demonstrated that TRPM8 activation acted on cytoskeleton dynamics and focal adhesions formation involving Rho GTPase signaling and, in particular, the inhibition of Cdc42 and Rac1 as well as that of the phosphorylation of ERK and FAK. Moreover, we applied lipid nanocapsules (LNCs) loaded with the TRPM8 agonist WS12, which limited the TRPM8–positive cells’ dissemination in metastatic sites with better efficiency than treatment with the agonist in its free form. Overall, our results confirm the anti–proliferative and anti–migratory role of TRPM8 in metastatic PCa cells and support the potential therapeutic use of TRPM8 to target PCa progression and PCa metastatic dissemination.

## Supplementary Materials

The following supporting information can be downloaded at: https://www.mdpi.com/article/10.3390/ijms23126672/s1.

## References

[1] Sung H., Ferlay J., Siegel R.L., Laversanne M., Soerjomataram I., Jemal A., Bray F. (2021). Global Cancer Statistics 2020: GLOBOCAN Estimates of Incidence and Mortality Worldwide for 36 Cancers in 185 Countries. CA A Cancer J. Clin..

[2] Bubendorf L., Schöpfer A., Wagner U., Sauter G., Moch H., Willi N., Gasser T.C., Mihatsch M.J. (2000). Metastatic patterns of prostate cancer: An autopsy study of 1589 patients. Hum. Pathol..

[3] Hanahan D., Weinberg R.A. (2011). Review Hallmarks of Cancer: The Next Generation. Cell.

[4] Flourakis M., Prevarskaya N. (2009). Insights into Ca^2+^ homeostasis of advanced prostate cancer cells. Biochim. Biophys. Acta-Mol. Cell Res..

[5] Monteith G.R., McAndrew D., Faddy H.M., Roberts-Thomson S.J. (2007). Calcium and cancer: Targeting Ca^2+^ transport. Nat. Rev. Cancer.

[6] Roderick H.L., Cook S.J. (2008). Ca^2+^ signalling checkpoints in cancer: Remodelling Ca^2+^ for cancer cell proliferation and survival. Nat. Rev. Cancer.

[7] Thebault S., Flourakis M., Vanoverberhe K., Vandermoere F., Roudbaraki M., Lehen’kyi V., Slomianny C., Beck B., Mariot P., Bonnal J. (2006). Differential Role of Transient Receptor Potential Channels in Ca^2+^ Entry and Proliferation of Prostate Cancer Epithelial Cells. Cancer Res..

[8] Pla A.F., Gkika D. (2013). Emerging role of TRP channels in cell migration: From tumor vascularization to metastasis. Front. Physiol..

[9] Prevarskaya N., Zhang L., Barritt G. (2007). TRP channels in cancer. Biochim. Biophys. Acta-Mol. Basis Dis..

[10] Prevarskaya N., Skryma R., Shuba Y. (2018). Ion channels in cancer: Are cancer hallmarks oncochannelopathies?. Physiol. Rev..

[11] Bai V.U., Murthy S., Chinnakannu K., Muhletaler F., Tejwani S., Barrack E.R., Kim S.H.O., Menon M., Reddy G.P.V. (2009). Androgen regulated TRPM8 expression: A potential mRNA marker for metastatic prostate cancer detection in body fluids. Int. J. Oncol..

[12] Schmidt U., Fuessel S., Koch R., Baretton G.B., Lohse A., Tomasetti S., Unversucht S., Froehner M., Wirth M.P., Meye A. (2006). Quantitative multi-gene expression profiling of primary prostate cancer. Prostate.

[13] Yang Z.-H., Wang X.-H., Wang H.-P., Hu L.-Q. (2009). Effects of TRPM8 on the proliferation and motility of prostate cancer PC-3 cells. Asian J. Androl..

[14] Zhu G., Wang X., Yang Z., Cao H., Meng Z., Wang Y., Chen D. (2011). Effects of TRPM8 on the proliferation and angiogenesis of prostate cancer PC-3 cells in vivo. Oncol. Lett..

[15] Wang Y., Wang X., Yang Z., Zhu G., Chen D., Meng Z. (2012). Menthol inhibits the proliferation and motility of prostate cancer DU145 cells. Pathol. Oncol. Res..

[16] Zhang L., Barritt G.J. (2004). Evidence that TRPM8 is an androgen-dependent Ca^2+^ channel required for the survival of prostate cancer cells. Cancer Res..

[17] Gkika D., Flourakis M., Lemonnier L., Prevarskaya N. (2010). PSA reduces prostate cancer cell motility by stimulating TRPM8 activity and plasma membrane expression. Oncogene.

[18] Genova T., Grolez G.P., Camillo C., Bernardini M., Bokhobza A., Richard E., Scianna M., Lemonnier L., Valdembri D., Munaron L. (2017). TRPM8 inhibits endothelial cell migration via a nonchannel function by trapping the small GTPase Rap1. J. Cell Biol..

[19] Valero M., Morenilla-Palao C., Belmonte C., Viana F. (2011). Pharmacological and functional properties of TRPM8 channels in prostate tumor cells. Pflugers Arch..

[20] Valero M.L., de Queiroz F.M., Stühmer W., Viana F., Pardo L.A. (2012). TRPM8 Ion Channels Differentially Modulate Proliferation and Cell Cycle Distribution of Normal and Cancer Prostate Cells. PLoS ONE.

[21] Grolez G.P., Hammadi M., Barras A., Gordienko D., Slomianny C., Völkel P., Angrand P.O., Pinault M., Guimaraes C., Potier-Cartereau M. (2019). Encapsulation of a TRPM8 Agonist, WS12, in Lipid Nanocapsules Potentiates PC3 Prostate Cancer Cell Migration Inhibition through Channel Activation. Sci. Rep..

[22] Millar F.R., Janes S.M., Giangreco A. (2017). Epithelial cell migration as a potential therapeutic target in early lung cancer. Eur. Respir. Rev..

[23] Tsavaler L., Shapero M.H., Morkowski S., Laus R. (2001). Trp-p8, a novel prostate-specific gene, is up-regulated in prostate cancer and other malignancies and shares high homology with transient receptor potential calcium channel proteins. Cancer Res..

[24] Henshall S.M., Afar D.E.H., Hiller J., Horvath L.G., Quinn D.I., Rasiah K.K., Gish K., Willhite D., Kench J.G., Gardiner-Garden M. (2003). Survival analysis of genome-wide gene expression profiles of prostate cancers identifies new prognostic targets of disease relapse. Cancer Res..

[25] Bidaux G., Flourakis M., Thebault S., Zholos A., Beck B., Gkika D., Roudbaraki M., Bonnal J.L., Mauroy B., Shuba Y. (2007). Prostate cell differentiation status determines transient receptor potential melastatin member 8 channel subcellular localization and function. J. Clin. Investig..

[26] Bidaux G., Roudbaraki M., Merle C., Crépin A., Delcourt P., Slomianny C., Thebault S., Bonnal J.L., Benahmed M., Cabon F. (2005). Evidence for specific TRPM8 expression in human prostate secretory epithelial cells: Functional androgen receptor requirement. Endocr. Relat. Cancer.

[27] Grolez G.P., Gordiendko D.V., Clarisse M., Hammadi M., Desruelles E., Fromont G., Prevarskaya N., Slomianny C., Gkika D. (2019). TRPM8-androgen receptor association within lipid rafts promotes prostate cancer cell migration. Cell Death Dis..

[28] Grolez G.P., Gkika D. (2016). TRPM8 Puts the Chill on Prostate Cancer. Pharmaceuticals.

[29] Thebault S., Lemonnier L., Bidaux G., Flourakis M., Bavencoffe A., Gordienko D., Roudbaraki M., Delcourt P., Panchin Y., Shuba Y. (2005). Novel role of cold/menthol-sensitive transient receptor potential melastatine family member 8 (TRPM8) in the activation of store-operated channels in LNCaP human prostate cancer epithelial cells. J. Biol. Chem..

[30] Borrelli F., Pagano E., Romano B., Panzera S., Maiello F., Coppola D., De Petrocellis L., Buono L., Orlando P., Izzo A.A. (2014). Colon carcinogenesis is inhibited by the TRPM8 antagonist cannabigerol, a Cannabis-derived non-psychotropic cannabinoid. Carcinogenesis.

[31] Wang Y., Yang Z., Meng Z., Cao H., Zhu G., Liu T., Wang X. (2013). Knockdown of TRPM8 suppresses cancer malignancy and enhances epirubicin-induced apoptosis in human osteosarcoma cells. Int. J. Biol. Sci..

[32] Du G.-J., Li J.-H., Liu W.-J., Liu Y.-H., Zhao B., Hou X.-D., Qi X.-X., Duan Y.-J., Li H.-R., Li H. (2013). The combination of TRPM8 and TRPA1 expression causes an invasive phenotype in lung cancer. Tumor Biol..

[33] Liu T., Fang Z., Wang G., Shi M., Wang X., Jiang K., Yang Z., Cao R., Tao H., Wang X. (2016). Anti-tumor activity of the TRPM8 inhibitor BCTC in prostate cancer DU145 cells. Oncol. Lett..

[34] Wertz I.E., Dixit V.M. (2000). Characterization of calcium release-activated apoptosis of LNCaP prostate cancer cells. J. Biol. Chem..

[35] Bidaux G., Beck B., Zholos A., Gordienko D., Lemonnier L., Flourakis M., Roudbaraki M., Borowiec A.S., Fernández J., Delcourt P. (2012). Regulation of activity of transient receptor potential melastatin 8 (TRPM8) channel by its short isoforms. J. Biol. Chem..

[36] Peng M., Wang Z., Yang Z., Tao L., Liu Q., Yi L., Wang X. (2015). Overexpression of short TRPM8 variant α promotes cell migration and invasion, and decreases starvation-induced apoptosis in prostate cancer LNCaP cells. Oncol. Lett..

[37] Rojas R., Ruiz W.G., Leung S.M., Jou T.S., Apodaca G. (2001). Cdc42-dependent modulation of tight junctions and membrane protein traffic in polarized Madin-Darby canine kidney cells. Mol. Biol. Cell.

[38] Takaishi K., Sasaki T., Kotani H., Nishioka H., Takai Y. (1997). Regulation of cell-cell adhesion by Rac and Rho small G proteins in MDCK cells. J. Cell Biol..

[39] Braga V.M.M., Del Maschio A., Machesky L., Dejana E. (1999). Regulation of Cadherin Function by Rho and Rac: Modulation by Junction Maturation and Cellular Context. Mol. Biol. Cell.

[40] Wang M., Zhao J., Zhang L., Wei F., Lian Y., Wu Y., Gong Z., Zhang S., Zhou J., Cao K. (2017). Role of tumor microenvironment in tumorigenesis. J. Cancer.

[41] Nagasaki T., Hara M., Nakanishi H., Takahashi H., Sato M., Takeyama H. (2014). Interleukin-6 released by colon cancer-associated fibroblasts is critical for tumour angiogenesis: Anti-interleukin-6 receptor antibody suppressed angiogenesis and inhibited tumour-stroma interaction. Br. J. Cancer.

[42] De Petrocellis L., Ligresti A., Schiano Moriello A., Iappelli M., Verde R., Stott C.G., Cristino L., Orlando P., Di Marzo V. (2013). Non-THC cannabinoids inhibit prostate carcinoma growth in vitro and in vivo: Pro-apoptotic effects and underlying mechanisms. Br. J. Pharmacol..

[43] Duncan D., Stewart F., Frohlich M., Urdal D. (2009). Preclinical Evaluation of the Trpm8 Ion Channel Agonist D-3263 for Benign Prostatic Hyperplasia. J. Urol..

[44] Gkika D., Lemonnier L., Shapovalov G., Gordienko D., Poux C., Bernardini M., Bokhobza A., Bidaux G., Degerny C., Verreman K. (2015). TRP channel-associated factors are a novel protein family that regulates TRPM8 trafficking and activity. J. Cell Biol..

[45] Noren N.K., Liu B.P., Burridge K., Kreft B. (2000). p120 Catenin regulates the actin cytoskeleton via RHO family GTPases. J. Cell Biol..

[46] Gkika D., Lolignier S., Grolez G.P., Bavencoffe A., Shapovalov G., Gordienko D., Kondratskyi A., Meleine M., Prival L., Chapuy E. (2020). Testosterone-androgen receptor: The steroid link inhibiting TRPM8-mediated cold sensitivity. FASEB J..

[47] Solaro R., Chiellini F., Battisti A. (2010). Targeted Delivery of Protein Drugs by Nanocarriers. Materials.

[48] Yallapu M.M., Khan S., Maher D.M., Ebeling M.C., Sundram V., Chauhan N., Ganju A., Balakrishna S., Gupta B.K., Zafar N. (2014). Anti-cancer activity of curcumin loaded nanoparticles in prostate cancer. Biomaterials.

[49] Huynh N.T., Passirani C., Saulnier P., Benoit J.P. (2009). Lipid nanocapsules: A new platform for nanomedicine. Int. J. Pharm..

[50] Driffort V., Gillet L., Bon E., Marionneau-Lambot S., Oullier T., Joulin V., Collin C., Pagès J.C., Jourdan M.L., Chevalier S. (2014). Ranolazine inhibits NaV1.5-mediated breast cancer cell invasiveness and lung colonization. Mol. Cancer.

[51] Rasband W. (2012). ImageJ.

[52] Van Duinen V., Heuvel A.V.D., Trietsch S.J., Lanz H.L., van Gils J., van Zonneveld A.J., Vulto P., Hankemeier T. (2017). 96 Perfusable Blood Vessels To Study Vascular Permeability In Vitro. Sci. Rep..

