# Supplementary material for: TRPM8 as an Anti–Tumoral Target in Prostate Cancer Growth and Metastasis Dissemination
Source: Int J Mol Sci. 2022 Jun 15;23(12):6672. doi: 10.3390/ijms23126672 (PMC9224463; doi:10.3390/ijms23126672)
Supplement: Supplementary file 1 [file ijms-23-06672-s001.zip › ijms-1737941-supplementary.pdf]

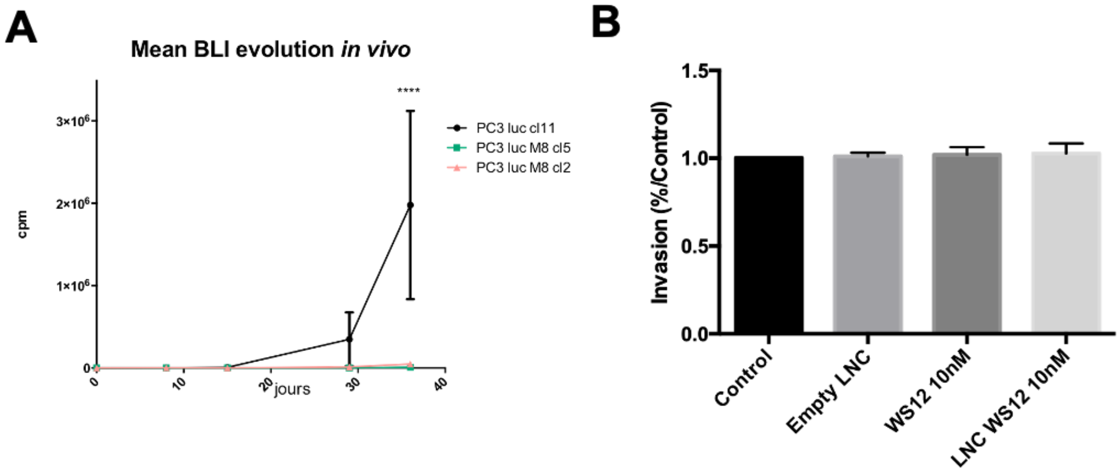

Figure S1. A) Quantification of bioluminescence 5 weeks after PC3-luc or PC3-M8-luc intravenous injection on the mice tail (n= 6 mice/condition; statistical significance: **** = P <0.0001, ordinary one-way ANOVA with *post-hoc* Bonferroni test). B) Invasion assay using Matrigel® transwell with PC3 cells. Data are normalized to the control and express as mean ± SEM (n=3 independent experiments at least; statistical significance: RM one-way ANOVA with *post-hoc* Tukey's test).
